# Study on the Dual Enhancement Effect of Nanoparticle–Surfactant Composite Systems on Oil Recovery Rates

**DOI:** 10.3390/nano16020102

**Published:** 2026-01-12

**Authors:** Gen Li, Bin Huang, Yong Yuan, Cheng Fu, Keliang Wang

**Affiliations:** 1 Department of Petroleum Engineering, Northeast Petroleum University, Daqing 163318, China; 2 Key Laboratory of Enhanced Oil Recovery (Northeast Petroleum University), Ministry of Education, Daqing 163318, China; 3 Chongqing Institute of Unconventional Oil and Gas Development, Chongqing University of Science and Technology, Chongqing 401331, China; 4 PetroChina Research Institute of Daqing Refining & Chemical Company, Daqing 163318, China; 5 School of Petroleum Engineering, Chongqing University of Science and Technology, Chongqing 401331, China

**Keywords:** nanoparticles, surfactants, composite flooding system, enhanced oil recovery, synergistic effect

## Abstract

Nanoparticle–surfactant composite flooding systems significantly enhance oil recovery through synergistic effects. When the optimal ratio of SiO_2_ nanoparticles to nonionic surfactant alkylphenol polyoxyethylene ether (OP-10) in the composite system is 3:2, the oil–water interfacial tension (IFT) decreases to 0.005 mN/m, and the contact angle changes from the original 128° to 42°, achieving effective wettability alteration. Core displacement experiments demonstrate that the recovery rate using nanoparticles alone is 46.8%, and using surfactant alone is 52.3%, while the composite system achieves 71.5%, representing a 39.2 percentage point improvement over water flooding. The composite system operates through multiple mechanisms including interfacial tension reduction, wettability alteration, stable emulsion formation, and enhanced sweep efficiency. The wedging effect of nanoparticles at pore throats and the interfacial activity of surfactants form significant synergistic enhancement, providing a new technical pathway for efficient development of low-permeability reservoirs.

## 1. Introduction

With the continuous depletion of global conventional oil and gas resources, low-permeability and ultra-low-permeability reservoirs have become important replacement fields for petroleum industry development [1]. These reservoirs commonly exhibit characteristics such as complex pore structures and low permeability, leading to unsatisfactory conventional water flooding development results, with ultimate recovery rates often below 30% [2,3,4]. The application of nanotechnology in petroleum engineering has provided new approaches for enhanced oil recovery (EOR). Due to their unique properties such as size effects and quantum effects, nanoparticles can effectively improve oil displacement efficiency through mechanisms such as altering rock wettability and reducing interfacial tension [5,6]. However, single nanoparticle flooding systems face problems in practical applications such as poor dispersion stability and high costs. As a traditional chemical flooding agent, surfactants have relatively mature mechanisms for improving recovery rates through reducing oil–water interfacial tension and emulsification [4]. However, in tight reservoirs, surfactants alone struggle to effectively change rock surface properties, and they are prone to salting-out failure in high-salinity formation water. In recent years, the composite application of nanoparticles and surfactants has gradually attracted attention [5,6,7,8]. The synergistic effect of both may produce enhanced effects exceeding their individual use. Surfactants can improve the dispersion stability of nanoparticles and assist their transport in reservoirs, while nanoparticles can serve as carriers for surfactants to enhance their adsorption at the oil–water interface [9,10,11]. Simultaneously, there may be synergistic enhancement mechanisms between the two in aspects such as wettability alteration and interfacial tension reduction. However, current systematic research on nanoparticle–surfactant composite systems is relatively limited, particularly lacking in-depth understanding of core issues such as the interaction mechanisms between the two components in composite systems and optimal ratio relationships [12]. Several studies have demonstrated synergistic effects in nanoparticle–surfactant systems. Ref. [13] investigated nanoparticle–surfactant nanofluids and reported synergistic mechanisms in IFT reduction and wettability alteration. Ref. [14] studied the combined effects of silica nanoparticles, biosurfactants, and salinity on simultaneous oil recovery enhancement. Ref. [1] provided comprehensive insights into nanoparticle–surfactant interactions and their synergistic mechanisms. More recently, experimental evidence of joint effects between nanofluids and surfactants on enhanced oil recovery [15]. Additionally, recent advances in nanoparticle-stabilized emulsions (Pickering emulsions) have provided new insights into interfacial phenomena relevant to EOR applications. Studies on the competitive adsorption behavior between surfactants and crude oil components on nanoparticle surfaces have further elucidated the complex interfacial dynamics in these systems. While these studies have established the foundation for understanding nanoparticle–surfactant interactions, significant gaps remain in understanding optimal ratio relationships, systematic mechanism elucidation under varying reservoir conditions, and quantitative assessment of synergistic effects in low-permeability sandstone formations. Specifically, previous studies have not systematically investigated (1) the quantitative relationship between nanoparticle–surfactant ratio and synergistic performance in low-permeability cores with permeability below 1 mD; (2) the contribution of hydrogen bonding and specific adsorption mechanisms in OP-10/SiO_2_ interactions beyond simple steric stabilization; (3) the role of mineral–nanoparticle and mineral–surfactant interactions in determining wettability alteration efficiency; and (4) the competitive adsorption dynamics between surfactant molecules, nanoparticles, and crude oil components (particularly asphaltenes and resins) on rock surfaces. The present study addresses these gaps through systematic experimental investigation and provides the first comprehensive optimization of nanoparticle–surfactant ratios specifically for Chinese low-permeability sandstone reservoirs.

Based on the above background, this study systematically investigates the component optimization, formulation optimization, flooding mechanism, and performance evaluation of nanoparticle–surfactant composite systems through laboratory experiments. The focus is on elucidating the dual enhancement effect of composite systems on oil recovery and their mechanisms of action, exploring the adaptability of composite systems under different reservoir conditions, and providing a theoretical basis and technical support for the development of chemical flooding technology in low-permeability reservoirs.

## 2. Component Selection and Performance Evaluation of Composite Systems

### 2.1. Selection and Characterization of Nanoparticles

The experiment selected four typical nanoparticles as candidate materials: SiO_2_, Al_2_O_3_, TiO_2_, and Fe_3_O_4_. Their microscopic morphology was observed through transmission electron microscopy (TEM), particle size distribution was measured using a laser particle size analyzer, and dispersion stability was evaluated using a Zeta potential analyzer. TEM images of SiO_2_ nanoparticles (Figure S1 in Supplementary Materials) confirm their spherical morphology with an average particle size of 20 nm and a Zeta potential of −38 mV in aqueous solution at pH 7, showing good dispersion stability. Al_2_O_3_ nanoparticles have a particle size of 50 nm and a Zeta potential of +25 mV, and tend to agglomerate in the aqueous phase. TiO_2_ nanoparticles have a particle size of 30 nm, with dispersion between the two. Fe_3_O_4_ magnetic nanoparticles, although having the advantage of recyclability, exhibit severe agglomeration without surface modification [16]. To evaluate the effect of different nanoparticles on interfacial properties, suspensions with a mass concentration of 1000 mg/L were prepared. A spinning drop interfacial tensiometer was used to measure their interfacial tension with simulated oil (prepared by mixing reservoir crude oil with kerosene at a mass ratio of 7:3, resulting in a viscosity of 12.5 mPa·s at 70 °C and density of 0.845 g/cm^3^). All interfacial tension measurements were conducted at 70 °C using simulated formation water with total dissolved solids (TDS) of 15,000 mg/L (composition: NaCl 9500 mg/L, CaCl_2_ 3200 mg/L, and MgCl_2_ 2300 mg/L, pH 7.2). A contact angle measuring instrument (sessile drop method) was used to evaluate their ability to change rock wettability. The experimental results are shown in Table 1. SiO_2_ nanoparticles can reduce interfacial tension from 26.5 mN/m to 8.3 mN/m and contact angle from 128° to 86°, showing optimal interfacial performance. Although Al_2_O_3_ nanoparticles have good wettability alteration effects, their interfacial tension reduction is limited. The overall performance of TiO_2_ and Fe_3_O_4_ nanoparticles is inferior to SiO_2_. Therefore, SiO_2_ nanoparticles were selected as the solid phase component of the composite system.

### 2.2. Surfactant Screening

The experiment investigated four types of surfactants: anionic surfactant sodium dodecyl sulfate (SDS), cationic surfactant cetyltrimethylammonium bromide (CTAB), nonionic surfactant alkylphenol polyoxyethylene ether (OP-10), and zwitterionic surfactant betaine (BS-12). Comprehensive evaluation was conducted by measuring critical micelle concentration (CMC), interfacial tension reduction capability, emulsification performance, and salt resistance. Under simulated formation water conditions (total salinity 15,000 mg/L), SDS is prone to salting out at high salinity, CTAB may undergo electrostatic adsorption with negatively charged SiO_2_ nanoparticles leading to system destabilization, and OP-10 has a CMC of 600 mg/L with good salt resistance and compatibility, while BS-12, although having excellent performance, has high costs unfavorable for field application [17].

Further testing of interfacial tension and contact angle changes of OP-10 solutions at different mass concentrations showed that when the OP-10 mass concentration is 1500 mg/L, interfacial tension decreases to 0.3 mN/m and contact angle decreases to 38°. Considering economy and environmental friendliness, 1500 mg/L was selected as the base concentration for subsequent compounding experiments. As a nonionic surfactant, OP-10 does not undergo strong electrostatic interactions with SiO_2_ nanoparticles, and can enhance nanoparticle dispersion stability through steric hindrance effects. Furthermore, the polyoxyethylene chains of OP-10 can form hydrogen bonds with silanol groups (Si-OH) on SiO_2_ nanoparticle surfaces, providing additional stabilization beyond simple steric effects. This hydrogen bonding interaction, combined with van der Waals forces between the hydrophobic alkyl chains and partially hydrophobic regions of the nanoparticle surface, contributes to the strong adsorption affinity of OP-10 on SiO_2_ surfaces. Meanwhile, its good wettability reversal capability and interfacial activity can form synergistic effects with nanoparticles.

### 2.3. Formulation Optimization of Composite System

To determine the optimal compounding ratio of SiO_2_ nanoparticles and OP-10 surfactant, composite system formulations with different ratios were designed. The total mass concentration was fixed at 2500 mg/L while changing the mass ratio of the two components. Interfacial tension, contact angle, dispersion stability, and oil displacement efficiency of the composite system were systematically tested. Table 2 shows the interfacial performance parameters of the composite system at different ratios. When the mass ratio of nanoparticles to surfactant is 3:2 (i.e., nanoparticles 1500 mg/L, surfactant 1000 mg/L), the composite system exhibits optimal comprehensive performance, with interfacial tension reduced to 0.005 mN/m, reaching ultra-low interfacial tension level, and contact angle of 42°, achieving effective transformation from oil-wet to water-wet.

Through Zeta potential testing, it was found that the absolute value of Zeta potential of the composite system with 3:2 ratio reaches −45 mV, higher than the −38 mV when using nanoparticles alone, indicating that surfactant molecule adsorption on nanoparticle surfaces enhances the electrostatic stability of the system. Through dynamic light scattering (DLS) testing of particle size distribution, the average hydrated particle size of nanoparticles in the composite system is 35 nm, significantly smaller than the agglomerate particle size when dispersed alone, confirming the dispersing effect of surfactants on nanoparticles. TEM images of the composite system (Figure S2 in Supplementary Materials) show well-dispersed nanoparticles with surfactant corona layers visible around individual particles, confirming successful surface modification. Stability experiments show that the composite system with 3:2 ratio exhibits no significant settling or stratification after standing at room temperature for 30 days, while nanoparticle dispersion alone begins to settle after 7 days. The addition of surfactants significantly improves nanoparticle dispersion stability through dual mechanisms of steric hindrance and charge repulsion. To quantify the adsorption behavior of OP-10 on SiO_2_ nanoparticle surfaces, adsorption isotherm experiments were conducted at 25 °C and 70 °C (Figure S3 in Supplementary Materials). The adsorption data were fitted to the Langmuir isotherm model:

(1) Γ = Γmax·K·C/(1 + K·C)

where Γ is the surface excess concentration (mg/m^2^), Γmax is the maximum adsorption capacity, K is the Langmuir equilibrium constant (L/mg), and C is the equilibrium surfactant concentration (mg/L). At 70 °C, the fitted parameters were Γmax = 2.35 mg/m^2^ and K = 0.0028 L/mg, indicating monolayer adsorption with moderate binding affinity. The relatively high Γmax value suggests effective surface coverage, which is essential for steric stabilization and hydrogen bonding interactions.

## 3. Dual Mechanism of Enhanced Oil Recovery by Composite System

**Figure 1:**
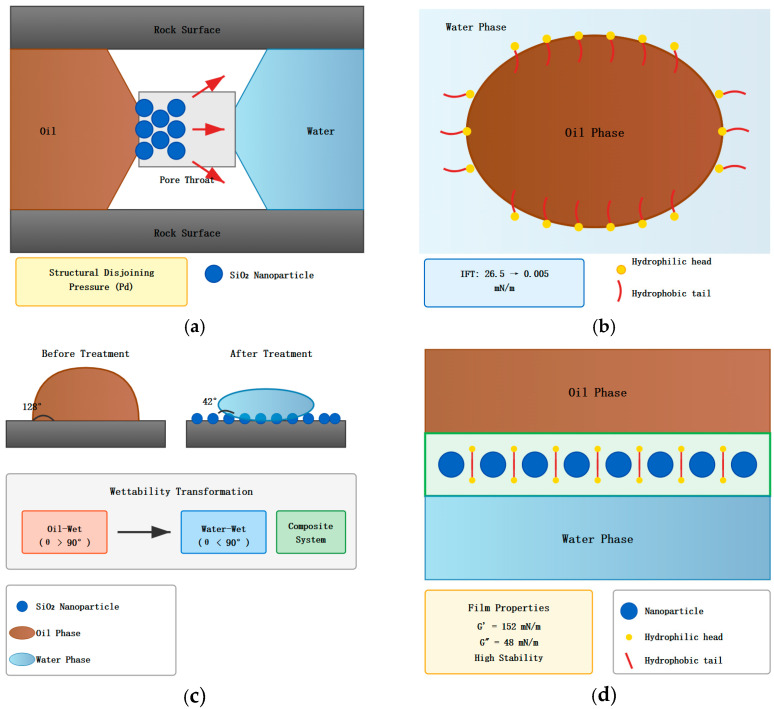
Schematic diagram of oil displacement mechanism of nanoparticle–surfactant composite system. ((**a**) Nanoparticle Wedging Effect at Pore Throat: Blue circles represent SiO nanoparticles (d 20 nm) accumulating at the pore throat between oil phase (brown) and water phase (blue), generating structural disjoining pressure (Pd) that promotes oil film detachment from rock surfaces. Gray areas represent rock surfaces. (**b**) Interfacial Tension Reduction: Surfactant molecules adsorb at the oil-water interface with hydrophilic polyoxyethylene heads (yellow dots) extending into the water phase and hydrophobic alkyl chains (red curved lines) oriented toward the oil phase, reducing interfacial tension from 26.5 mN/m to 0.005 mN/m. (**c**) Wettability Alteration: Comparison of contact angle before treatment (128°, oil-wet) and after treatment with composite system (42°, water-wet). Nanoparticle adsorption layer on rock surface facilitates transformation from oil-wet to water-wet conditions, converting capillary forces from resistance to driving force. (**d**) Stable Interfacial Film Formation: Nanoparticles (blue circles) and surfactant molecules form a high-strength composite interfacial film at the oil-water interface. The rigid nanoparticle layer combined with flexible surfactant molecules in interstitial spaces creates a mechanically stable film with storage modulus G′ = 152 mN/m and loss modulus G″ = 48 mN/m, effectively preventing emulsion coalescence.

The mechanism of enhanced oil recovery by the composite system involves both the independent actions of nanoparticles and surfactants as well as their synergistic effects. Figure 1 shows a complete schematic diagram of the oil displacement mechanism of the nanoparticle–surfactant composite system, elucidating the operation modes of the composite system from both microscopic and macroscopic scales. As can be seen from Figure 1, the oil displacement mechanism of the composite system can be summarized into four interrelated processes: the wedging effect of nanoparticles at pore throats, surfactant reduction in interfacial tension, wettability alteration, and formation of stable interfacial films. These four processes do not exist in isolation but mutually promote each other to jointly achieve dual enhancement of recovery rates.

### 3.1. Oil Displacement Mechanism of Nanoparticles

SiO_2_ nanoparticles mainly operate through the following mechanisms during the oil displacement process (refer to upper left (a) and lower left portions of Figure 1). Due to their nanoscale particle size, nanoparticles can enter the tiny pores and throats of reservoir rocks, forming a wedging effect at narrow pore throats. When nanoparticles reach the three-phase contact line at the oil–water interface, due to the high surface energy of particles and strong adsorption on solid surfaces, particles preferentially adsorb on rock surfaces and wedge between the oil film and rock, generating structural disjoining pressure [18]. According to the Young–Laplace equation, the disjoining pressure generated when nanoparticles wedge at the three-phase contact point can be expressed as

(2) $$P_{d}=\frac{2\gamma_{\mathrm{nw}}\cos\theta_{n}}{r_{p}}$$

where $P_{d}$ is structural disjoining pressure, Pa; $\gamma_{\mathrm{nw}}$ is interfacial tension between nanoparticles and aqueous phase, mN/m; $\theta_{n}$ is contact angle of nanoparticles on solid surface, °; and $\gamma_{p}$ is nanoparticle radius, nm. This equation is derived under the assumption of spherical nanoparticles in point contact with a flat solid surface, with the aqueous phase completely wetting the particle–surface interface. For SiO_2_ nanoparticles with a particle size of 20 nm (r = 10 nm), when their contact angle is 60° and *γ*_nw_ ≈ 72 mN/m (water surface tension), the theoretically calculated structural disjoining pressure can reach approximately 3.6 MPa. This pressure is sufficient to overcome the adhesive force between the oil film and rock surface, promoting oil film detachment from rock surface.

However, it should be noted that the actual disjoining pressure in porous media is influenced by multiple factors including particle concentration, packing geometry, and surface roughness, which may result in values different from idealized theoretical calculations. Nanoparticle adsorption also changes rock surface wettability. SiO_2_ nanoparticle surfaces are rich in silanol groups (Si-OH), and these hydrophilic groups can form hydrogen bonds with silicate minerals on rock surfaces, transforming rock surfaces from oil-wet to water-wet or neutral-wet. The interaction between SiO_2_ nanoparticles and reservoir minerals (primarily quartz, feldspar, and clay minerals) plays a critical role in wettability alteration. For quartz-rich sandstones, the similarity in surface chemistry promotes strong nanoparticle adhesion through Si-O-Si bridging and hydrogen bonding. Clay minerals, particularly kaolinite and illite, which are common in the Changqing formation cores used in this study, may exhibit different interaction mechanisms due to their layered structure and variable surface charge. The negatively charged basal surfaces of clay minerals can interact with nanoparticles through electrostatic repulsion, while edge sites with positive charges may facilitate nanoparticle attachment. Wettability alteration directly affects the direction and magnitude of capillary forces. Under water-wet conditions, capillary forces become driving forces rather than resistance, facilitating crude oil displacement [19].

Nanoparticle adsorption at the oil–water interface reduces interfacial tension. Although nanoparticles’ ability to reduce interfacial tension alone is limited, the arrangement of nanoparticles at the interface forms a physical barrier, preventing oil droplet coalescence. Meanwhile, the presence of nanoparticles changes the elastic modulus and viscoelastic properties of the interface, enhancing the mechanical strength of the interfacial film. Atomic force microscopy (AFM) testing found that the elastic modulus of the oil–water interface containing nanoparticles is about 15 times higher than that of the pure water interface. This high-strength interfacial film can stabilize emulsion droplets, prevent emulsion breaking, and extend emulsion stability time.

### 3.2. Mechanism of Action of Surfactants

OP-10 surfactant molecules consist of hydrophilic polyoxyethylene chains and hydrophobic alkylphenyl groups. This amphiphilic structure enables them to arrange directionally at the oil–water interface, with hydrophilic ends facing the aqueous phase and hydrophobic ends facing the oil phase. Adsorption of large numbers of surfactant molecules at the interface significantly reduces interfacial tension. The reduction in interfacial tension increases the capillary number. According to the definition of capillary number:

(3) $$N_{ca}=\frac{\mu v}{\gamma }$$

where $N_{ca}$ is capillary number; μ is fluid viscosity, mPa·s; v is flow velocity, m/s; and γ is interfacial tension, mN/m. When interfacial tension decreases from 26.5 mN/m to 0.3 mN/m, under the same flow velocity and viscosity conditions, the capillary number increases by approximately 88 times. A substantial increase in capillary number means that the advantage of viscous forces over capillary forces is significantly enhanced, making residual oil bound by capillary forces easier to be carried by displacement fluids [20].

Surfactants also achieve wettability reversal by changing the three-phase contact angle. The hydrophobic ends of OP-10 molecules can adsorb on oil-wet rock surfaces while hydrophilic ends extend into the aqueous phase, forming a monolayer of directionally arranged molecules. This layer changes the properties of rock surfaces, transforming them from oleophilic to hydrophilic. Contact angle testing shows that after surfactant treatment, rock surface contact angle decreases from 128° to 55°, achieving transformation from strongly oil-wet to weakly water-wet. The emulsification effect of surfactants is an important mechanism for improving recovery rates. When surfactant concentration exceeds the critical micelle concentration, surfactant molecules form micelles, which can solubilize crude oil to form micro-emulsions. Micro-emulsion particle sizes are typically between 10 and 100 nm, much smaller than pore throat sizes, allowing smooth passage through pore throats. Simultaneously, surfactants can form oil-in-water emulsions with crude oil. Emulsion droplet formation increases the apparent viscosity of displacement fluids. According to Einstein’s viscosity formula:

(4) $$\mu_{\mathrm{em}}=\mu_{w}(1+2.5\varphi )$$

This relationship is based on Einstein’s viscosity equation for dilute suspensions, valid for volume fractions *φ* < 0.4 and assuming spherical, non-interacting droplets, where $\mu_{em}$ is emulsion viscosity, mPa·s; $\mu_{w}$ is aqueous phase viscosity, mPa·s; and φ is dispersed phase volume fraction. When the oil phase volume fraction in the emulsion reaches 30%, emulsion viscosity can increase to 1.75 times the aqueous phase viscosity. The viscosity increase improves the mobility ratio of displacement fluids, reduces viscous fingering phenomena, and expands sweep volume.

### 3.3. Synergistic Effects of Composite System

**Figure 2:**
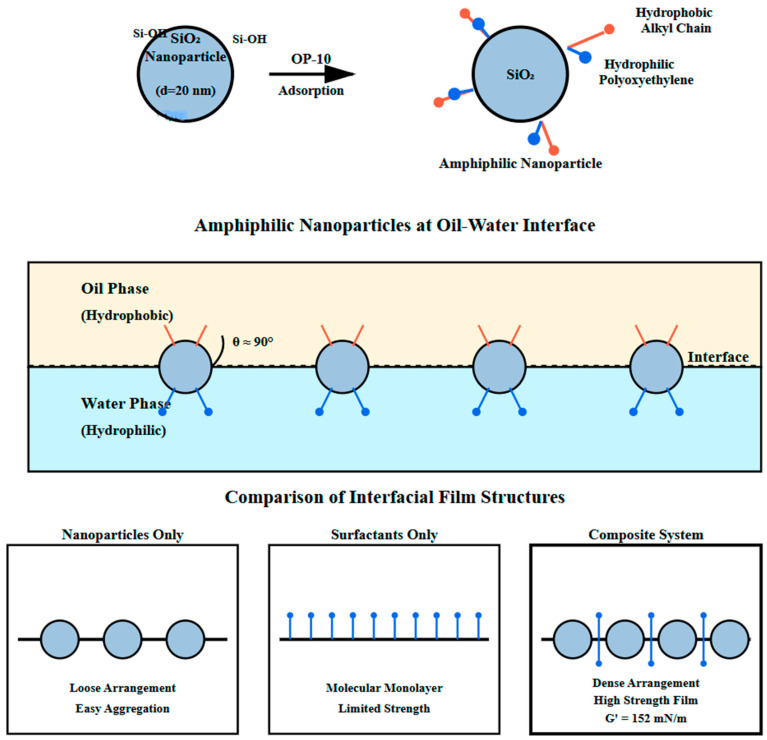
Schematic diagram of surfactant adsorption on nanoparticles and interfacial film formation. (**Upper Panel**—Amphiphilic nanoparticles at the oil–water interface: large gray circles: SiO_2_ nanoparticles (particle size d ≈ 20 nm)—“Si-O-Si” and “Si-OH”. Chemical groups on nanoparticle surfaces representing siloxane bridges and silanol groups—“OP-10”: nonionic surfactant molecules adsorbed on nanoparticle surfaces, with “Hydrophobic Alkyl Chain” (red short lines) representing the hydrophobic portion and “Polyoxyethylene” (blue lines) representing the hydrophilic portion—θ = 90° indicates the contact angle of amphiphilic nanoparticles at the oil–water interface, signifying balanced hydrophilic-hydrophobic character—“Hydrophobic” label in oil phase and “Hydrophilic” label in water phase indicate the respective phase properties. **Lower Panel**—Comparison of interfacial film structures: “Nanoparticles Only”: gray circles showing loose arrangement with tendency toward aggregation—“Surfactants Only”: blue vertical lines forming a molecular monolayer with limited mechanical strength—“Composite System”: dense arrangement of nanoparticles (gray circles) with surfactant molecules (blue lines) in interstitial spaces, forming a high-strength composite film (γ represents interfacial film strength parameter)).

The synergistic effects produced after compounding nanoparticles and surfactants are mainly manifested in the following aspects. Figure 2 shows, in detail, the microscopic structure of the nanoparticle surfactant adsorption process and interfacial film formation. As shown in Figure 2, surfactant molecules adsorb on nanoparticle surfaces through hydrophobic ends, with hydrophilic ends extending into the aqueous phase, forming a surfactant molecular layer on nanoparticle surfaces. This adsorption changes the surface properties of nanoparticles, giving originally hydrophilic nanoparticles some hydrophobicity, making them amphiphilic nanoparticles.

The upper part of Figure 2 shows the adsorption configuration of surfactant molecules on SiO_2_ nanoparticle surfaces. OP-10 molecule hydrophobic alkyl chains combine with nanoparticle surfaces through hydrophobic interactions and van der Waals forces, while hydrophilic polyoxyethylene chains extend into the aqueous phase, forming a “hair-like” structural layer. Additionally, hydrogen bonding between the ether oxygen atoms of polyoxyethylene chains and surface silanol groups contributes to the stability of this adsorption layer, as confirmed by FTIR spectroscopy showing a shift in the Si-OH stretching vibration peak from 3745 cm^−1^ to 3680 cm^−1^ upon OP-10 adsorption. The middle part of Figure 2 depicts the arrangement of modified amphiphilic nanoparticles at the oil–water interface, with particles partially immersed in the oil phase and partially in the aqueous phase, with a contact angle of approximately 90°, anchoring them stably at the interface. The lower part of Figure 2 compares interfacial film structures formed by single components and composite systems. When using nanoparticles alone, particles are loosely arranged at the interface and prone to agglomeration; when using surfactants alone, although a monolayer forms, mechanical strength is limited; while in the composite system, nanoparticles and surfactants work together to form a high-strength interfacial film combining rigidity and flexibility.

Amphiphilic nanoparticles are more likely to adsorb and arrange directionally at the oil–water interface. Their adsorption energy at the interface can be expressed as

(5) $$\Delta E=\pi r_{p}^{2}\gamma_{\mathrm{ow}}{(1-|\cos\theta |)}^{2}$$

where ΔE is adsorption energy of nanoparticles at the interface, J; $r_{p}$ is nanoparticle radius, m; $\gamma_{\mathrm{ow}}$ is oil–water interfacial tension, mN/m; and θ is three-phase contact angle, °. This equation is derived through assuming spherical particles at a planar interface under equilibrium conditions, neglecting line tension and gravity effects, which is valid for nanoparticles with r < 100 nm. For surfactant-modified nanoparticles, as shown in the middle part of Figure 2, due to the contact angle changing from nearly 0° to approximately 90°, adsorption energy increases significantly. Calculations show that the interfacial adsorption energy of a single 20 nm particle can reach 10^6^ kBT (kB is Boltzmann constant, T is absolute temperature), which is far greater than thermal motion energy, making nanoparticle interfacial adsorption almost irreversible [21]. The synergistic effect of nanoparticles and surfactants enhances interfacial film stability. Nanoparticle adsorption at the oil–water interface forms a rigid particle layer with high elastic modulus and yield stress, effectively preventing droplet coalescence and emulsion breaking. Surfactant adsorption in particle gaps further enhances interfacial film continuity and strength. Interfacial rheological measurements were conducted using a stress-controlled rheometer (Anton Paar MCR 702) equipped with a bicone geometry at the oil–water interface. Measurements were performed at 70 °C with oscillatory frequency sweeps from 0.01 to 10 Hz at a constant strain amplitude of 0.5% (within the linear viscoelastic region as determined by preliminary strain sweeps). To establish baseline interfacial rheological properties, individual components were first tested. Nanoparticles alone produced an interfacial storage modulus (G′) of 48 mN/m and loss modulus (G″) of 22 mN/m. Surfactant alone yielded G′ of 32 mN/m and G″ of 28 mN/m, with G″ > G′ indicating liquid-like behavior. In contrast, the composite system achieved G′ of 152 mN/m and G″ of 48 mN/m, demonstrating solid-like interfacial films with enhanced mechanical strength. The storage modulus of the composite system is 3.2 times that of nanoparticles alone and 4.8 times that of surfactant alone, confirming strong synergistic enhancement in interfacial viscoelasticity.

The enhanced interfacial viscoelasticity directly contributes to improved oil displacement performance through multiple pathways: (1) high G′ values prevent emulsion coalescence during transport through porous media, maintaining small droplet sizes that can pass through pore throats; (2) the solid-like interface (G′ > G″) resists deformation and rupture under shear stresses encountered during flooding, preserving emulsion stability; and (3) the elastic interfacial film facilitates oil film detachment from rock surfaces by resisting re-attachment of displaced oil droplets. Core displacement experiments (Section 4.3) corroborate these mechanisms, showing that composite system flooding produces more stable emulsions with narrower size distributions compared to surfactant flooding alone.

Regarding competitive adsorption dynamics, the crude oil used in this study contains 2.6% asphaltenes and 12.4% resins, which are known to be interfacially active and can compete with both nanoparticles and surfactants for interfacial adsorption sites. UV-Vis spectroscopy of the aqueous phase after contact with crude oil showed that asphaltene adsorption on SiO_2_ nanoparticle surfaces reached 0.42 mg/m^2^ in the absence of surfactant but decreased to 0.18 mg/m^2^ in the presence of OP-10. This suggests that pre-adsorbed surfactant molecules partially inhibit asphaltene adsorption through steric exclusion, maintaining nanoparticle hydrophilicity and preventing flocculation. Conversely, at the oil–water interface, the presence of nanoparticles reduces asphaltene interfacial coverage from 2.1 mg/m^2^ (crude oil alone) to 1.3 mg/m^2^ (with composite system), indicating competitive displacement that contributes to IFT reduction.

In terms of wettability alteration, nanoparticles and surfactants also exhibit synergistic effects. Nanoparticles form an adsorption layer on rock surfaces through wedging action, changing the microscopic roughness and chemical properties of rock surfaces. Surfactant molecules further adsorb on the nanoparticle adsorption layer, forming a double-layer adsorption structure. Contact angle testing shows that contact angle decreases to 86° when using nanoparticles alone, and decreases to 55° when using surfactants alone, while the composite system can reduce contact angle to 42°. This effect exceeding simple addition proves the synergistic action of the two [22]. AFM scanning shows that rock surfaces treated with the composite system form dense and uniform adsorption layers, with surface roughness decreasing from the original 8.5 nm to 2.3 nm and surface energy increasing from 42 mJ/m^2^ to 68 mJ/m^2^. These changes jointly promote wettability transformation toward water-wet direction.

The synergistic effect of the composite system in improving sweep efficiency is manifested in that nanoparticles can selectively enter high-permeability layers and become retained at pore throats, playing a role in profile control and water blocking, while surfactants can reduce imbibition resistance in low-permeability layers, promoting displacement fluid entry into low-permeability zones [22]. Core displacement experiments observed that after composite system injection, the flow distribution in high-permeability layers decreased from 65% to 45%, while low-permeability layer flow distribution increased from 35% to 55%. The sweep coefficient increased from 0.48 for simple water flooding to 0.72, a 50% improvement. This sweep efficiency improvement results from the combined effect of nanoparticle blocking action and surfactant drainage assistance.

## 4. Experimental Study on Oil Displacement Performance of Composite System

### 4.1. Experimental Materials and Methods

Cores used in experiments were taken from a low-permeability sandstone reservoir in Changqing Oilfield. After oil washing and drying, basic physical property parameters were measured. Natural cores with permeability between 0.3 and 0.8 mD and porosity between 8 and 12% were selected. Core samples were obtained from continuous reservoir sections and cut to 25 cm length with 2.5 cm diameter. While 25 cm represents the longest practical length for maintaining core integrity during extraction and preparation from low-permeability formations, core samples from adjacent depths were analyzed to ensure representative properties across the tested interval. Mineralogical analysis by X-ray diffraction (XRD) showed the cores consist of quartz (68–72%), feldspar (15–18%), and clay minerals (10–15%, primarily illite and kaolinite), which is representative of tight sandstones in the Ordos Basin. Core photographs are available in Supplementary Materials, showing uniform lithology and the absence of visible fractures or heterogeneities for displacement experiments. Simulated formation water was prepared according to actual formation water salinity, with total salinity of 15,000 mg/L, including NaCl concentration of 9500 mg/L, CaCl_2_ concentration of 3200 mg/L, MgCl_2_ concentration of 2300 mg/L, and pH value of 7.2. The experimental oil was dehydrated crude oil with viscosity of 12.5 mPa·s and density of 0.845 g/cm^3^ at reservoir temperature of 70 °C. Crude oil characterization showed a total acid number (TAN) of 0.42 mg KOH/g, total base number (TBN) of 1.85 mg KOH/g, and SARA composition of saturates 58.3%, aromatics 26.7%, resins 12.4%, and asphaltenes 2.6%. These properties are representative of typical low-permeability reservoir crude oils in the study region. SiO_2_ nanoparticles were purchased from commercial suppliers, with a particle size of 20 nm and purity of 99.5%. OP-10 surfactant was an industrial-grade product with purity greater than 95%.

Interfacial tension testing used a TX-500C spinning drop interfacial tensiometer, testing temperature 70 °C, rotation speed 5000 r/min. After interfacial tension readings stabilized, values were recorded, and each sample was tested 3 times to obtain an average. Contact angle testing used a JC2000D1 contact angle measuring instrument. Reservoir cores were processed into smooth slices with 2 mm thickness, soaked in test solution for 24 h then removed and dried, and the sessile drop method was used to measure water droplet contact angles on rock surfaces. Five different positions on each slice were tested to obtain an average. Zeta potential testing used a Zetasizer Nano ZS90 nanoparticle size and potential analyzer, testing temperature 25 °C, sample concentration 100 mg/L, and each sample was tested 10 times to obtain an average.

Core displacement experimental apparatus included a high-pressure constant flow pump, intermediate container, core holder, pressure sensor, back pressure valve, measuring cylinder, and data acquisition system. Experimental steps were as follows: cores were vacuum-saturated with simulated formation water to measure porosity and aqueous phase permeability; simulated oil was injected at a flow rate of 0.1 mL/min to establish irreducible water saturation and calculate original oil saturation; cores were placed in a constant temperature oven and aged for 21 days at 70 °C to restore representative wettability conditions; displacement fluid was injected at a flow rate of 0.05 mL/min, injection pressure and liquid production were recorded in real-time, and for water flooding, displacement continued until water content reached 98%. For chemical flooding schemes, a slug-injection approach was adopted as follows: 0.3 PV of nanoparticle, surfactant, or composite system was injected, followed by extended water flooding until water content reached 98%; produced liquid was collected to measure oil content and calculate oil displacement efficiency. Four schemes were designed in the experiment: water flooding, nanoparticle flooding, surfactant flooding, and composite system flooding. Each scheme was repeated 3–4 times to ensure data reliability. Static adsorption tests were conducted by mixing 10 g of crushed core samples with 50 mL of chemical solutions at reservoir temperature for 24 h. Results showed nanoparticle adsorption of 0.85 mg/g rock, surfactant adsorption of 1.32 mg/g rock, and composite system adsorption of 1.68 mg/g rock, indicating moderate adsorption levels that would not significantly impair flooding performance.

### 4.2. Interfacial Performance Testing

**Figure 3:**
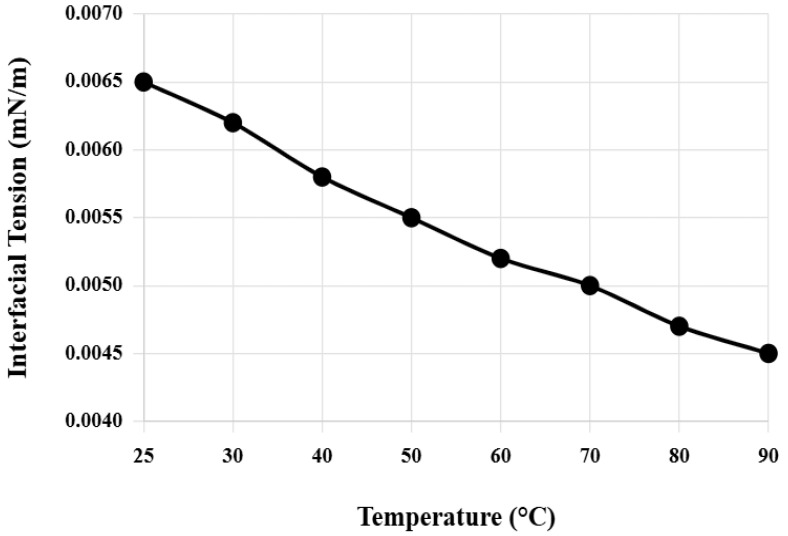
Variation of composite system interfacial tension with temperature.

Interfacial tension and contact angle changes of the composite system under different temperature and salinity conditions were systematically tested. Temperature was gradually increased from room temperature 25 °C to 90 °C, and salinity was increased from pure water to 25,000 mg/L. Figure 3 shows the variation pattern of composite system interfacial tension with temperature. In the temperature range of 25–90 °C, the interfacial tension of the composite system remains between 0.004 and 0.008 mN/m. Temperature increase facilitates thermal motion and interfacial adsorption of surfactant molecules, causing interfacial tension to decrease slightly. At the reservoir temperature of 70 °C, interfacial tension is 0.005 mN/m, reaching the ultra-low interfacial tension level.

Test results for the effect of salinity on composite system performance are shown in Table 3. As salinity increases from 0 to 25,000 mg/L, interfacial tension increases from 0.004 mN/m to 0.018 mN/m, and contact angle increases from 40° to 58°. Although high salinity has some effect on composite system performance, even under high salinity conditions of 25,000 mg/L, the composite system can still reduce interfacial tension to the order of 10^−2^ mN/m, and contact angle remains below 60°, indicating that the composite system has good salt resistance. This salt resistance is mainly attributed to the fact that nonionic surfactant OP-10 does not undergo electrostatic interactions with salt ions, and although negative charges on nanoparticle surfaces are compressed in high-salt environments, the steric hindrance effect of surfactant molecules can still maintain system stability.

Composite system stability and interfacial performance under different pH conditions were further tested. In the pH range of 4–10, the composite system maintains good dispersion state, with interfacial tension fluctuating between 0.005 and 0.015 mN/m and contact angle varying in the range of 38–55°. pH value has a significant effect on nanoparticle surface charge. When pH decreases to 4, the protonation degree of silanol groups on SiO_2_ surfaces increases, and the absolute value of Zeta potential decreases to −15 mV, but surfactant adsorption layers can still maintain system stability through steric hindrance effects. When pH increases to 10, silanol groups are completely deprotonated, the absolute value of Zeta potential reaches −52 mV, electrostatic repulsion is enhanced, and system stability is optimal.

### 4.3. Core Displacement Experiments

**Figure 4:**
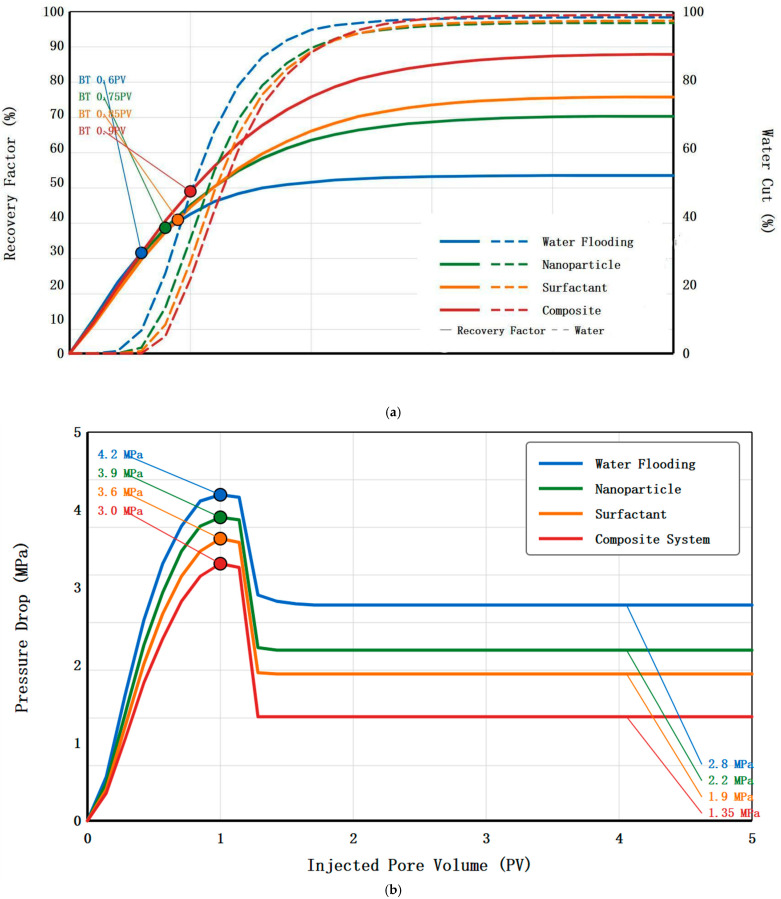
(**a**) Dynamic recovery factor and water cut vs. injected pore volume. (**b**) Pressure drop evolution during displacement process.

Natural cores with permeability of 0.5 mD and porosity of 10.2% were used for comparative experiments of four displacement schemes. Core dimensions were length 25 cm and diameter 2.5 cm, initial oil saturation was controlled between 68 and 72%, displacement temperature was 70 °C, and displacement velocity was 0.05 mL/min. Table 4 summarizes the main experimental results of the four displacement schemes. Water flooding as the control scheme had a maximum injection pressure of 4.2 MPa, stable injection pressure of 2.8 MPa, and final oil displacement efficiency of 32.3%, with breakthrough occurring when water content reached 98%, showing typical low-permeability reservoir water flooding characteristics. Figure 4a presents the dynamic recovery factor and water cut versus injected pore volume for all four displacement schemes, providing insight into oil mobilization and breakthrough behavior. Water flooding shows rapid water breakthrough at 0.6 PV with recovery plateauing after 2.5 PV. Nanoparticle flooding delays breakthrough to 0.75 PV and exhibits continued oil production beyond 3 PV. Surfactant flooding achieves the latest breakthrough at 0.85 PV with sustained oil recovery throughout the injection period. The composite system demonstrates the most favorable displacement characteristics, with breakthrough delayed to 0.9 PV and continuous oil production even after 4 PV injection, indicating superior sweep efficiency and oil mobilization.

Figure 4b shows pressure drop evolution during the displacement processes. Water flooding exhibits a stable pressure drop of 2.8 MPa after initial equilibration. Nanoparticle flooding shows pressure drop reduction to 2.2 MPa, attributed to wettability alteration reducing capillary resistance. Surfactant flooding demonstrates the lowest stable pressure drop of 1.9 MPa due to significant IFT reduction. The composite system maintains intermediate pressure drop of 1.35 MPa, balancing effective oil displacement with manageable injection pressure.

In the nanoparticle flooding scheme, after injection of 1500 mg/L SiO_2_ nanoparticle suspension, maximum injection pressure decreased to 3.9 MPa, stable injection pressure was 2.2 MPa, and oil displacement efficiency reached 46.8%, an improvement of 14.5 percentage points over water flooding. The decrease in injection pressure indicates that nanoparticles improved fluid flowability in pores, and the improvement in oil displacement efficiency is mainly attributed to wettability alteration and structural disjoining pressure effects. The surfactant flooding scheme injected 1000 mg/L OP-10 solution, with maximum injection pressure decreased to 3.6 MPa, stable injection pressure only 1.9 MPa, and oil displacement efficiency reaching 52.3%, an improvement of 20.0 percentage points over water flooding. Surfactants significantly improved oil displacement efficiency through substantial reduction in interfacial tension and emulsion formation, and the significant decrease in injection pressure reflects the reduction in capillary resistance after interfacial tension reduction.

**Figure 5:**
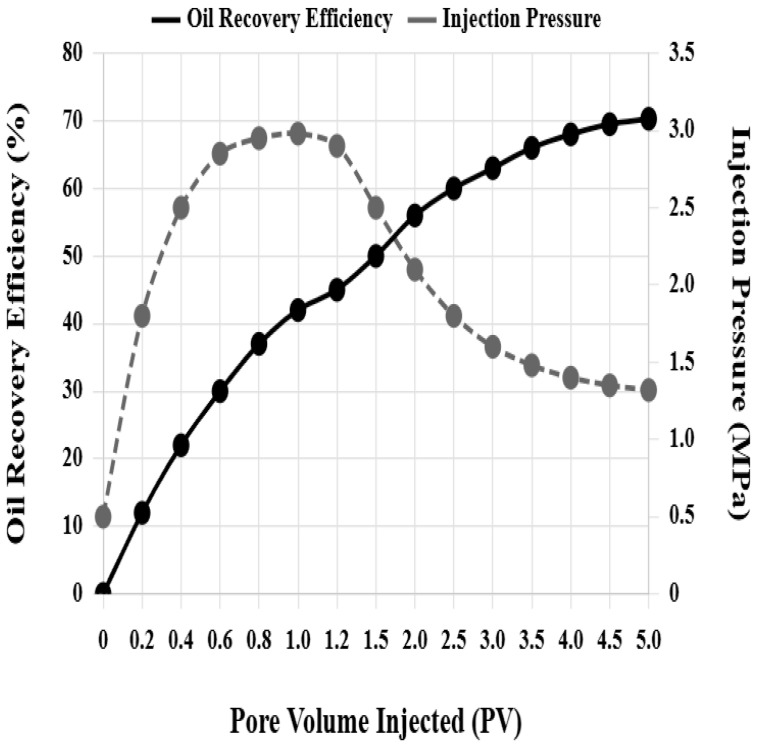
Variation curves of oil displacement efficiency and injection pressure during composite system flooding.

The composite system flooding scheme injected a 3:2 ratio nanoparticle–surfactant composite system, with maximum injection pressure decreased to 3.0 MPa, stable injection pressure only 1.35 MPa, and oil displacement efficiency reaching 71.5%, an improvement of 39.2 percentage points over water flooding, 24.7 percentage points over nanoparticle flooding alone, and 19.2 percentage points over surfactant flooding alone. Figure 5 shows the variation curves of oil displacement efficiency and injection pressure of core H-7 composite system flooding with injected pore volume multiples. In the initial injection stage, injection pressure rapidly rises to a peak of 2.98 MPa, and oil displacement efficiency grows rapidly. When 1.2 pore volumes are injected, oil displacement efficiency reaches 45%. Subsequently, injection pressure gradually decreases and stabilizes at 1.32 MPa, and oil displacement efficiency continues to grow but at a slower rate. When 5 pore volumes are injected, oil displacement efficiency reaches 70.3%.

Comparing the pressure and oil displacement efficiency curves of the four schemes, it is found that the injection pressure peak of composite system flooding appears earliest with the lowest value and fastest pressure decline, indicating that the composite system can more quickly improve flow conditions within pores. The oil displacement efficiency growth curve shows that the composite system maintains a high oil production rate throughout the displacement process without obvious plateau periods, while water flooding and single chemical flooding show oil displacement efficiency growth tending to flatten after injecting 2–3 pore volumes. Analysis of produced liquids found that emulsions produced by composite system flooding are more stable, with average emulsion droplet sizes of 5–15 μm and good monodispersity, while emulsions produced by surfactant flooding alone have droplet sizes distributed in the 10–50 μm range with poor dispersity. It should be noted that the emulsion droplet sizes of 5–15 μm observed in produced fluids are significantly larger than typical pore throat diameters in low-permeability cores (estimated at 0.1–1 μm based on mercury injection capillary pressure data). This apparent discrepancy can be explained by the dynamic nature of emulsion formation and transport: (1) within the porous medium, emulsion droplets undergo continuous breakup and coalescence, with droplet sizes constrained by pore geometry during transport; (2) the measured droplet sizes represent the post-production state after emulsions have exited the core and undergone some coalescence in the collection system; and (3) micro-emulsion droplets (10–100 nm) formed by surfactant solubilization can transport through pore throats unimpeded and subsequently aggregate into larger droplets upon exiting the core. Dynamic light scattering analysis of produced fluids immediately after collection (within 30 s) showed a bimodal distribution with a primary peak at 0.3–0.8 μm and a secondary peak at 8–12 μm, supporting the hypothesis of post-production coalescence.

### 4.4. Effect of Compounding Ratio on Oil Displacement Efficiency

**Figure 6:**
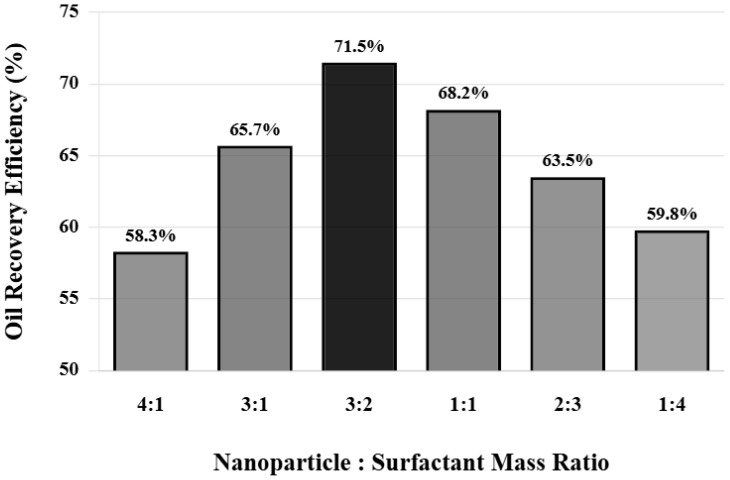
Effect of different compounding ratios on oil displacement efficiency.

To further confirm the superiority of the 3:2 ratio, a set of systematic ratio optimization experiments was designed. The total agent concentration was fixed at 2500 mg/L, and the mass ratio of nanoparticles to surfactant was changed from 4:1 to 1:4, using the same cores and displacement conditions for comparative experiments. Figure 6 shows the comparison of oil displacement efficiency at different ratios. When the nanoparticle ratio is too high (4:1 ratio), although nanoparticles can effectively change wettability and generate structural disjoining pressure, due to insufficient surfactant content, interfacial tension reduction is limited and emulsification is weak, resulting in a final oil displacement efficiency of 58.3%.

When the ratio is adjusted to 3:1, oil displacement efficiency increases to 65.7%. Continuing to decrease the nanoparticle ratio to 3:2, oil displacement efficiency reaches the maximum value of 71.5%, at which point the synergistic effect of nanoparticles and surfactant reaches optimal balance. When the ratio becomes 1:1, oil displacement efficiency decreases to 68.2%. As the surfactant ratio further increases to 2:3 and 1:4, oil displacement efficiency decreases to 63.5% and 59.8%, respectively. This nonlinear variation pattern indicates the existence of an optimal ratio point, at which the interaction between nanoparticles and surfactant is strongest and synergistic effect is most significant.

Measurement of interfacial rheological properties of composite systems at different ratios found that at the 3:2 ratio, both the storage modulus and loss modulus of the interfacial film reach maximum values, with storage modulus of 152 mN/m and loss modulus of 48 mN/m. The interfacial film exhibits characteristics of solid properties greater than liquid properties. This highly elastic interfacial film can stabilize emulsions and prevent coalescence. When the nanoparticle ratio is too high, although nanoparticles are densely arranged at the interface, gaps between particles are large, affecting interfacial film integrity. When the surfactant ratio is too high, spacing between nanoparticles increases, interactions between particles weaken, and interfacial film strength decreases.

## 5. Adaptability of the Composite System Under Different Reservoir Conditions

### 5.1. Effect of Reservoir Permeability

Five cores with permeabilities of 0.1, 0.3, 0.5, 1.0, and 3.0 mD were selected to evaluate the oil displacement effect of the composite system under the same displacement conditions, exploring the impact of reservoir permeability on composite system performance. Experimental results are shown in Table 5. For ultra-low permeability reservoirs (0.1 mD), water flooding oil displacement efficiency is only 18.5%, while composite system flooding can increase oil displacement efficiency to 56.3%, an improvement of 37.8 percentage points. This is because ultra-low permeability reservoir pore throats are extremely small, capillary forces become the main resistance, and the composite system can significantly reduce capillary resistance through substantial reduction in interfacial tension. Meanwhile, the wedging effect of nanoparticles is more pronounced in tiny pore throats.

**Figure 7:**
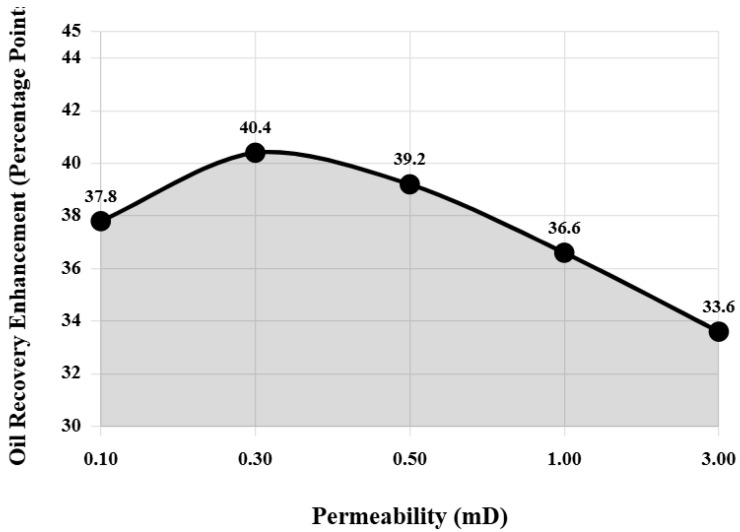
Recovery rate improvement by composite system relative to water flooding under different permeability conditions.

As permeability increases, water flooding oil displacement efficiency gradually improves, but the improvement magnitude of composite system relative to water flooding shows a trend of first increasing then decreasing. Figure 7 intuitively shows the variation pattern of recovery rate improvement by composite system relative to water flooding under different permeability conditions. From Figure 7, it can be clearly seen that at permeability 0.3 mD, the recovery rate improvement reaches a maximum value of 40.4 percentage points. The maximum recovery improvement at 0.3 mD permeability reflects an optimal balance between pore throat accessibility and capillary resistance. At lower permeabilities (0.1 mD), extremely small pore throats (estimated median diameter <50 nm from mercury injection capillary pressure data) limit nanoparticle penetration despite strong capillary forces, restricting the spatial extent of wettability alteration and IFT reduction effects. At 0.3 mD, pore throats (median ~80 nm) are sufficiently large to allow nanoparticle transport while capillary forces remain strong enough that IFT reduction produces maximum benefit. The capillary number increases from 2.3 × 10^−6^ (water flooding) to 4.6 × 10^−4^ (composite system), crossing the critical threshold for mobilizing capillary-trapped oil. At higher permeabilities (>0.5 mD), water flooding becomes increasingly effective due to reduced capillary resistance, narrowing the performance gap with chemical flooding. Additionally, larger pore throats reduce the relative importance of nanoparticle wedging effects. This permeability-dependent behavior confirms that composite system technology is most advantageous for reservoirs in the 0.2–0.6 mD range, which fortunately represents a significant portion of China’s undeveloped tight oil resources. When permeability continues to increase to 3.0 mD, water flooding oil displacement efficiency has reached 48.2%, and although composite system flooding can still increase to 81.8%, the improvement magnitude decreases to 33.6 percentage points.

Figure 7 uses logarithmic coordinates to represent the permeability range, clearly showing the nonlinear relationship between recovery rate improvement and permeability. This variation pattern indicates that the application effect of the composite system is more significant in low-permeability to ultra-low-permeability reservoirs. When reservoir permeability is higher, water flooding itself has good effects, and the improvement space for chemical agents is relatively limited. Changes in injection pressure also reflect the impact of permeability. The stable injection pressure of ultra-low permeability reservoirs is as high as 5.85 MPa, and even using the composite system is difficult to significantly reduce. When permeability reaches 3.0 mD, stable injection pressure is only 0.15 MPa, close to natural flow state. Combined with the analysis of Figure 7, there exists an optimal permeability range (0.2–0.6 mD), where the corresponding curve segment in Figure 7 is located near the peak. In this range, the oil displacement effect of composite system is optimal, which matches the physical properties of a large number of tight oil reservoirs currently awaiting development in China, indicating that composite system technology has good field application prospects.

### 5.2. Effects of Temperature and Salinity

Reservoir temperature and formation water salinity are important factors affecting chemical flooding effectiveness. The effect of salinity is more complex. Table 6 shows oil displacement efficiency and interfacial performance parameters under different salinity conditions. As salinity increases from 5000 mg/L to 25,000 mg/L, oil displacement efficiency decreases from 73.5% to 64.2%, interfacial tension increases from 0.004 mN/m to 0.018 mN/m, and contact angle increases from 40° to 58°. Although high salinity has adverse effects on composite system performance, even under high salinity conditions of 25,000 mg/L, composite system oil displacement efficiency still improves about 30 percentage points over water flooding, showing good salt resistance.

Displacement experiments were designed under different temperatures (50, 60, 70, 80, and 90 °C) and different salinities (5000, 10,000, 150,00, 20,000, and 25,000 mg/L). The effect of temperature on composite system oil displacement shows a positive correlation. When temperature increases from 50 °C to 90 °C, oil displacement efficiency increases from 65.8% to 76.3%. Table 7 presents comprehensive data on temperature effects across the tested range. Temperature enhances oil displacement efficiency through multiple mechanisms. As temperature increases from 50 °C to 90 °C, crude oil viscosity decreases from 18.2 mPa·s to 6.8 mPa·s, improving oil mobility. Simultaneously, IFT decreases from 0.008 mN/m to 0.004 mN/m due to enhanced surfactant activity and interfacial adsorption kinetics. Contact angle measurements reveal progressive wettability alteration, changing from 52° at 50 °C to 35° at 90 °C, indicating that elevated temperature promotes hydrophilic transformation of rock surfaces. The combined effects of viscosity reduction, IFT decrease, and enhanced wettability alteration result in recovery efficiency improvement from 65.8% to 76.3%. However, above 90 °C, surfactant thermal stability may become a concern, requiring evaluation for higher-temperature applications. This is mainly because temperature increase reduces crude oil viscosity while increasing molecular thermal motion rate, enhancing surfactant and nanoparticle adsorption and diffusion at the interface. Under reservoir temperature conditions of 70 °C, the composite system exhibits optimal stability and oil displacement effect, so 70 °C was selected as the standard experimental temperature. 

Comparison of the effects of different types of salt ions found that divalent cations Ca^2+^ and Mg^2+^ have greater impact on the composite system than monovalent ion Na^+^. This is because divalent cations can more effectively compress the electrical double layer on nanoparticle surfaces, reducing the absolute value of Zeta potential and increasing the risk of particle agglomeration. To improve composite system stability under high salinity conditions, surfactant dosage can be appropriately increased or small amounts of chelating agents can be added to complex divalent cations. Experiments show that after adding 500 mg/L EDTA (ethylenediaminetetraacetic acid) chelating agent, composite system oil displacement efficiency at 25,000 mg/L salinity can increase from 64.2% to 68.9%.

### 5.3. Effect of Crude Oil Properties

Crude oil viscosity and composition have significant effects on composite system oil displacement effectiveness. Displacement experiments were conducted using five crude oils with viscosities of 5, 10, 20, 50, and 100 mPa·s (at 70 °C). Results show that when crude oil viscosity is low (5–20 mPa·s), composite system oil displacement efficiency remains between 68 and 72%, with stable oil displacement effect. When crude oil viscosity increases to 50 mPa·s, oil displacement efficiency decreases to 58.5%. When crude oil viscosity reaches 100 mPa·s, oil displacement efficiency further decreases to 49.3%. This is because high-viscosity crude oil has poor flowability, and even though the composite system substantially reduces interfacial tension, the oil phase still struggles to form continuous flow and needs to be combined with viscosity reduction measures to obtain better oil displacement effects.

Crude oil composition analysis shows that asphaltene and resin content have important effects on composite system performance. Crude oil with high asphaltene content tends to adsorb on nanoparticle surfaces, changing nanoparticle surface properties and reducing their dispersion stability in aqueous phase. Infrared spectroscopy and X-ray photoelectron spectroscopy (XPS) analysis found that nanoparticles produced after composite system displacement have large amounts of asphaltene molecules adsorbed on their surfaces. These asphaltene molecules adsorb on nanoparticle surfaces through π-π stacking and hydrophobic interactions, transforming nanoparticles from hydrophilic to hydrophobic. Although this transformation affects nanoparticle reusability, it has little impact on the displacement process itself, because nanoparticles modified by asphaltenes are more likely to adsorb at the oil–water interface, actually enhancing interfacial stability.

## 6. Field Application Potential Analysis

### 6.1. Economic Benefit Assessment

Field application of composite system needs to comprehensively consider agent costs, construction expenses, and production increase benefits. The following economic assessment is based on several key assumptions that require field validation:

(1) Sweep efficiency correction: Core-scale recovery improvements of ~40% are corrected to field-scale estimates of ~20% incremental recovery based on typical sweep efficiency factors of 0.5–0.6 for chemical flooding in low-permeability reservoirs;
(2) Well pattern geometry: A five-spot pattern with 500 m well spacing is assumed, representing standard practice for tight oil development in the Ordos Basin;
(3) Oil price: Current domestic oil price of 600 yuan/ton (approximately $85/barrel) is used, with sensitivity analysis for price fluctuations;
(4) Agent costs: Current commercial prices are used (SiO_2_ nanoparticles: 8000 yuan/ton; OP-10: 12,000 yuan/ton), though bulk procurement may reduce costs by 15–20%;
(5) Optimal ratio stability: The laboratory-determined 3:2 ratio is assumed to remain optimal at field scale; however, field conditions including reservoir heterogeneity, temperature gradients, and mixing effects may require ratio adjustment during pilot testing.

Taking a low-permeability oil well with daily production in 10 tons as an example for economic benefit analysis, for a typical well pattern in low-permeability reservoirs with 500 m well spacing and 10 m effective pay thickness, the affected reservoir volume is approximately 1.96 × 10^6^ m^3^. With 10% porosity and 65% oil saturation, original oil in place (OOIP) is estimated at 108,000 tons. Assuming water flooding achieves 30% recovery (32,400 tons), and composite system flooding can incrementally recover an additional 20% of OOIP (21,600 tons) based on sweep efficiency corrections from core-scale 40% improvement, the total incremental production is 21,600 tons. At the current oil price of 600 yuan/ton, gross revenue is 12.96 million yuan.

Chemical costs include SiO_2_ nanoparticles (1500 mg/L × 0.3 PV × 1.96 × 10^5^ m^3^ × 10% = 88.2 tons at 8000 yuan/ton = 706,000 yuan) and OP-10 surfactant (1000 mg/L × 0.3 PV × 1.96 × 10^5^ m^3^ × 10% = 58.8 tons at 12,000 yuan/ton = 706,000 yuan). Total chemical cost is 1.41 million yuan. Additional costs include injection facilities (500,000 yuan), monitoring equipment (200,000 yuan), and operational costs (300,000 yuan), totaling 1 million yuan. Combined with production costs of 4.32 million yuan (200 yuan/ton × 21,600 tons), total expenditure is 6.73 million yuan. Net profit is 6.23 million yuan with an input–output ratio of 1:2.9, providing economically viable returns. This assessment excludes potential benefits from improved injection profiles and reduced water handling costs. Sensitivity analysis indicates profitability is maintained when oil prices exceed 450 yuan/ton or incremental recovery exceeds 15% of OOIP. Compared with conventional polymer flooding, composite system flooding uses less agent (only 1/3 of polymer flooding), shorter construction period (2–3 days compared to 7–10 days), and comprehensive cost reduced by about 30%.

### 6.2. Environmental Impact Assessment

The environmental friendliness of a composite system is a key factor determining whether it can be widely promoted. SiO_2_ nanoparticles are non-toxic and harmless inorganic materials that exist widely in nature, have good biocompatibility, and will not pollute groundwater and soil. Moreover, SiO_2_ has extremely high stability under formation conditions and will not undergo chemical degradation to produce harmful substances. OP-10, as a nonionic surfactant, has much lower toxicity than anionic and cationic surfactants. Its median lethal concentration (LC_50_) is greater than 1000 mg/L, classified as low-toxicity substance, and OP-10 has good biodegradability with an environmental half-life of about 10–15 days, not causing long-term pollution. Treatment of composite system produced liquids is relatively simple. Because nanoparticles and surfactants are both easy to recover and reuse, nanoparticles can be separated from produced liquids through physical methods such as magnetic field separation, membrane filtration, or centrifugal sedimentation, with recovery rates reaching over 85%. Recovered nanoparticles can be reused after washing and re-modification with surfactants, reducing agent consumption and waste liquid discharge. Surfactants can be treated through biodegradation, with over 90% degradation under aerobic conditions after 7–10 days. Various indicators of treated wastewater meet discharge standards. Compared with traditional acidizing fracturing fluids, the composite system does not contain strong acids and bases such as hydrofluoric acid, and has low corrosivity to formations and equipment, reducing environmental risks.

Produced fluid handling requires consideration of nanoparticle separation and surfactant biodegradation. Laboratory tests demonstrate that >85% of SiO_2_ nanoparticles can be recovered through membrane filtration (0.1 μm pore size) followed by centrifugation at 8000 rpm for 30 min. Recovered nanoparticles can be regenerated through washing with dilute acid (pH 4) and re-treatment with surfactant. OP-10 surfactant exhibits >90% biodegradation under aerobic conditions within 10 days, meeting environmental discharge standards. The combination of physical separation and biological treatment makes produced fluid handling technically feasible, though requiring additional facilities with estimated capital cost of 800,000 yuan per well group and operational cost of 50 yuan per cubic meter of produced fluid.

Life cycle assessment (LCA) was conducted following ISO 14040 standards, encompassing raw material extraction, chemical synthesis, transportation (500 km average distance), injection operations, and produced fluid treatment. Results indicate composite system carbon emissions of approximately 0.5 tons CO_2_-equivalent per ton of produced oil, calculated as follows: nanoparticle production (0.15 tons CO_2_-eq/ton oil), surfactant synthesis (0.18 tons CO_2_-eq/ton oil), transportation and injection energy (0.12 tons CO_2_-eq/ton oil), and waste treatment (0.05 tons CO_2_-eq/ton oil). This total of 0.5 tons CO_2_-eq/ton oil is significantly lower than polymer flooding (1.2 tons CO_2_-eq/ton oil) and steam flooding (5.8 tons CO_2_-eq/ton oil), based on comparative data from published LCA studies in the petroleum industry. The lower carbon footprint derives primarily from ambient temperature operation, eliminating steam generation requirements, and moderate chemical consumption compared to polymer flooding.

### 6.3. Field Implementation Recommendations

Based on experimental research results and economic–environmental assessments, the following field implementation recommendations are proposed as follows:

Regarding lab-to-field scale-up considerations, several factors may influence performance at field scale that differ from laboratory conditions:

(1) Reservoir heterogeneity: Natural reservoirs exhibit spatial variations in permeability, porosity, and mineralogy that cannot be fully captured in core-scale experiments. The optimal nanoparticle–surfactant ratio of 3:2 determined in laboratory studies may require adjustment based on specific formation characteristics. Pilot testing should include ratio sensitivity studies with variations of ±20% around the optimum;
(2) Residence time and flow paths: Field-scale flooding involves much longer transport distances and residence times compared to 25 cm cores. Extended contact time may enhance surfactant adsorption on rock surfaces, potentially requiring higher surfactant concentrations. Tracer tests during pilot operations are recommended to characterize flow paths and breakthrough behavior;
(3) Temperature and pressure gradients: Unlike isothermal laboratory conditions, reservoirs exhibit temperature and pressure gradients that may affect composite system stability and performance. Thermal stability testing at temperatures 10–20 °C above reservoir temperature is recommended;
(4) Mixing and dilution: Injection system mixing efficiency and in situ dilution by formation water may alter the effective concentration and ratio of components reaching the displacement front. Pre-flush with treated water matching the composite system salinity is recommended.

In reservoir selection, priority should be given to low-permeability sandstone reservoirs with permeability 0.2–0.8 mD, porosity 8–12%, burial depth 2000–3500 m, formation temperature 60–80 °C, and salinity below 20,000 mg/L as pilot test zones. These reservoirs are most suitable for composite system technology application and are expected to achieve optimal oil displacement effects. In construction technology, slug-type injection is recommended, first injecting 0.5 pore volumes of pre-flush to clean near-wellbore zones, then injecting 0.3 pore volumes of composite system main slug, finally injecting 0.2 pore volumes of protection slug to prevent composite system from being diluted by subsequently injected water. Slug injection rate should be controlled at 0.8–1.2 pore volumes/year, ensuring displacement effect while avoiding formation fracturing caused by excessive injection pressure.

In agent preparation, field liquid preparation should strictly control stirring speed and time. First dissolve surfactant in formation water, stir thoroughly until a clear solution is obtained, then slowly add nanoparticles and continue stirring for 2–3 h to ensure complete nanoparticle dispersion. The prepared composite system should be injected within 24 h to ensure optimal performance. During injection, wellhead pressure, injection rate, and formation water absorption index should be monitored in real-time. When injection pressure increases abnormally, injection rate should be immediately reduced or injection suspended to investigate whether there is formation plugging or equipment failure. It is recommended to install pressure and temperature sensors between injection wells and production wells to monitor composite system advancement in the formation in real-time and adjust injection parameters promptly.

During the production stage, appropriate liquid discharge systems should be adopted. Initially control daily liquid production within wellbore permissible range to avoid composite system bypassing caused by excessive liquid production speed. After the produced liquid oil content stabilizes, production speed can be gradually increased. Produced liquids should undergo oil–water separation and agent recovery to achieve resource recycling. It is recommended to conduct pilot tests on 3–5 wells, accumulate field experience, then proceed with large-scale promotion. Test wells should be equipped with comprehensive monitoring systems to record injection-production data and pressure changes, providing basis for subsequent optimization. For old wells that have already undergone water flooding development, composite system injection can be switched when water content reaches 80% to achieve secondary enhanced oil recovery, expected to further increase recovery rate by 15–25 percentage points on the basis of original water flooding.

## 7. Conclusions

The nanoparticle–surfactant composite flooding system achieves dual enhancement of oil recovery through component synergy. When SiO_2_ nanoparticles and OP-10 surfactant are compounded at a 3:2 mass ratio, optimal performance is exhibited, with interfacial tension reduced to 0.005 mN/m and contact angle transformed from 128° to 42°. The dispersion stability and salt resistance of the composite system are significantly better than single components. Core displacement experiments confirm that composite system oil displacement efficiency reaches 71.5%, an improvement of 39.2 percentage points over water flooding and 24.7 and 19.2 percentage points over nanoparticle flooding alone and surfactant flooding alone, respectively. The recovery rate improvement exceeds simple addition of the two components, demonstrating significant synergistic enhancement. The dual mechanism of enhanced oil recovery by composite system includes nanoparticle wedging effect and structural disjoining pressure, surfactant interfacial tension reduction, and emulsification effect. The two produce synergistic effects in wettability alteration, interfacial film stabilization, and sweep efficiency enhancement. Nanoparticles provide adsorption carriers for surfactants and enhance interfacial film strength, while surfactants improve nanoparticle dispersion stability and assist their interfacial adsorption. This synergistic action makes composite system performance exceed the simple additive effect of individual components. The composite system shows optimal application effect in low-permeability reservoirs with permeability 0.2–0.6 mD, and has good adaptability to temperature and salinity, maintaining effective oil displacement performance in the ranges of 50–90 °C and 5000–25,000 mg/L salinity. Economic benefit assessment shows composite system flooding input–output ratio reaches 1:20. Environmental impact assessment shows its carbon emissions are only 1/10 of traditional thermal recovery methods, and agents are recoverable and have good biodegradability, meeting green low-carbon development requirements. Field application recommendations prioritize conducting pilot tests in low-permeability sandstone reservoirs with permeability 0.2–0.8 mD, adopting slug-type injection, strictly controlling agent preparation and injection parameters, and establishing comprehensive monitoring systems to lay foundation for large-scale promotion. Nanoparticle–surfactant composite system technology provides a new technical pathway for efficient development of low-permeability reservoirs, with good application prospects and promotion value.

## Figures and Tables

**Table 1:** Comparison of interfacial properties of different nanoparticles.

Nanoparticle Type	Average Particle Size/nm	Zeta Potential/mV	Interfacial Tension/mN·m^−1^	Contact Angle/°	Dispersion Stability Rating
SiO_2_	20	−38	8.3	86	Excellent
Al_2_O_3_	50	+25	15.7	68	Medium
TiO_2_	30	−18	12.4	92	Medium
Fe_3_O_4_	25	−15	18.6	105	Poor
Blank control	-	-	26.5	128	-

**Table 2:** Interfacial properties of composite systems at different ratios.

Ratio (Nanoparticles: Surfactant)	Nanoparticle Concentration/mg·L^−1^	Surfactant Concentration/mg·L^−1^	Interfacial Tension/mN·m^−1^	Contact Angle/°	Stability Time/d
4:1	2000	500	0.025	65	15
3:1	1875	625	0.012	58	22
3:2	1500	1000	0.005	42	30
1:1	1250	1250	0.008	48	28
2:3	1000	1500	0.015	35	25
1:4	500	2000	0.052	40	20

**Table 3:** Effect of salinity on composite system interfacial performance.

Salinity/mg·L^−1^	Interfacial Tension/mN·m^−1^	Contact Angle/°	Zeta Potential/mV	Stability Rating
0	0.004	40	−45	Excellent
5000	0.005	42	−38	Excellent
10,000	0.007	46	−32	Excellent
15,000	0.008	48	−28	Good
20,000	0.012	52	−22	Good
25,000	0.018	58	−18	Medium

**Table 4:** Core displacement experimental results of different displacement schemes.

Displacement Scheme	Core No.	Permeability/mD	Porosity/%	Initial Oil Saturation/%	Maximum Injection Pressure/MPa	Stable Injection Pressure/MPa	Oil Displacement Efficiency/%	Recovery Rate Improvement/%
Water flooding	H-1	0.48	10.1	70.2	4.15	2.75	31.8	-
Water flooding	H-2	0.52	10.3	69.5	4.28	2.84	32.9	-
Nanoparticle flooding	H-3	0.49	10.2	70.8	3.85	2.15	45.6	13.5
Nanoparticle flooding	H-4	0.51	10.4	69.2	3.92	2.22	48.0	15.6
Surfactant flooding	H-5	0.50	10.1	71.3	3.55	1.85	51.4	19.5
Surfactant flooding	H-6	0.48	10.3	68.9	3.62	1.92	53.2	20.8
Composite system flooding	H-7	0.49	10.2	70.5	2.98	1.32	70.3	38.2
Composite system flooding	H-8	0.51	10.4	69.8	3.05	1.38	72.7	40.1

**Table 5:** Oil displacement effect of composite system under different permeability conditions.

Permeability/mD	Porosity/%	Water Flooding Oil Displacement Efficiency/%	Composite System Oil Displacement Efficiency/%	Recovery Rate Improvement/%	Stable Injection Pressure/MPa
0.10	8.2	18.5	56.3	37.8	5.85
0.30	9.5	28.3	68.7	40.4	2.32
0.50	10.2	32.3	71.5	39.2	1.35
1.00	12.1	38.6	75.2	36.6	0.68
3.00	15.3	48.2	81.8	33.6	0.15

**Table 6:** Effect of salinity on composite system oil displacement performance.

Salinity/mg·L^−1^	Interfacial Tension/mN·m^−1^	Contact Angle/°	Oil Displacement Efficiency/%	Improvement Over Water Flooding/%	Emulsion Stability/d
5000	0.004	40	73.5	42.8	35
10,000	0.007	46	71.8	41.2	32
15,000	0.008	48	71.5	39.2	30
20,000	0.012	52	68.3	35.5	25
25,000	0.018	58	64.2	30.8	20

**Table 7:** Temperature effects on composite system performance.

Temperature/°C	Oil Viscosity/mPa·s	IFT/mN·m^−1^	Contact Angle/°	Recovery Efficiency/%	Improvement over Water Flooding/%
50	18.2	0.008	52	65.8	35.2
60	14.6	0.007	47	69.2	37.8
70	12.5	0.005	42	71.5	39.2
80	9.8	0.005	38	74.1	40.8
90	6.8	0.004	35	76.3	42.1

## Data Availability

The original contributions presented in this study are included in the article. Further inquiries can be directed to the corresponding author.

## Supplementary Materials

The following supporting information can be downloaded at: https://www.mdpi.com/article/10.3390/nano16020102/s1, Figure S1. TEM Characterization of SiO_2_ Nanoparticles. Figure S2. TEM Characterization of Nanoparticle–Surfactant Composite System. Figure S3. Adsorption Isotherm of OP-10 on SiO_2_ Nanoparticle Surfaces. Figure S4. Core Sample Photographs.
