# Supplementary material for: Study on the Dual Enhancement Effect of Nanoparticle–Surfactant Composite Systems on Oil Recovery Rates
Source: Nanomaterials (Basel). 2026 Jan 12;16(2):102. doi: 10.3390/nano16020102 (PMC12844195; doi:10.3390/nano16020102)
Supplement: Supplementary file 1 [file nanomaterials-16-00102-s001.zip › nanomaterials-4068409-supplementary materials.pdf]

Supplementary Materials

Study on the Dual Enhancement Effect of Nanoparticle–Surfactant Composite Systems on Oil Recovery Rates

Gen Li [1,2,†], Bin Huang [3,†], Yong Yuan [4], Cheng Fu [5] and Keliang Wang [1,2,*]

[1] Department of Petroleum Engineering, Northeast Petroleum University, Daqing 163318, China
[2] Key Laboratory of Enhanced Oil Recovery (Northeast Petroleum University), Ministry of Education, Daqing 163318, China
[3] Chongqing Institute of Unconventional Oil and Gas Development, Chongqing University of Science and Technology, Chongqing 401331, China
[4] PetroChina Research Institute of Daqing Refining & Chemical Company, Daqing 163318, China
[5] School of Petroleum Engineering, Chongqing University of Science and Technology, Chongqing 401331, China
* Correspondence: wkl626@outlook.com (K.W.)
† These authors contributed equally to this work.

Figure S1. TEM Characterization of SiO_2 Nanoparticles

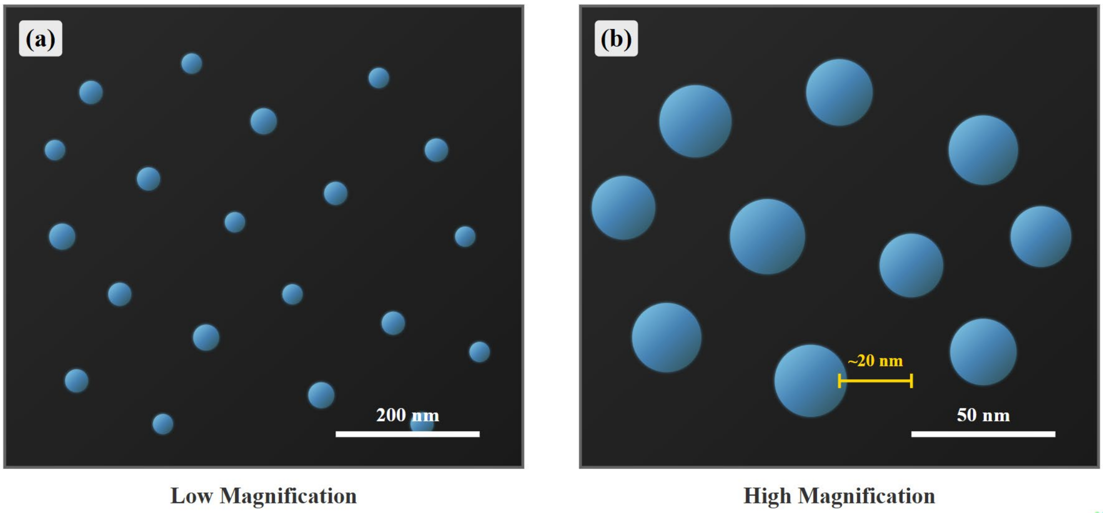

Figure S1. Transmission electron microscopy (TEM) images of SiO_2 nanoparticles used in this study. (a) Low-magnification TEM image showing the overall morphology and distribution of nanoparticles; (b) High-magnification TEM image confirming the spherical morphology with an average particle size of approximately 20 nm. The nanoparticles exhibit uniform size distribution with minimal agglomeration in the as-received state. Scale bars: (a) 200 nm; (b) 50 nm.

Figure S2. TEM Characterization of Nanoparticle–Surfactant Composite System

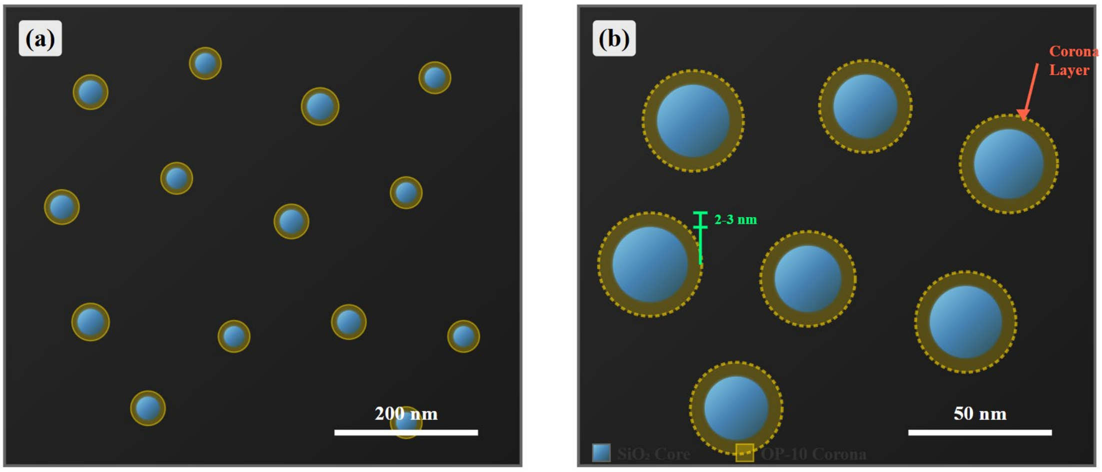

Figure S2. Transmission electron microscopy (TEM) images of the SiO_2 nanoparticle–OP-10 surfactant composite system at the optimal 3:2 mass ratio. (a) Low-magnification image demonstrating well-dispersed nanoparticles without significant agglomeration; (b) High-magnification image showing individual nanoparticles with visible surfactant corona layers (indicated by arrows) surrounding the particle surfaces, confirming successful surface modification through surfactant adsorption. The corona layer thickness is estimated to be approximately 2–3 nm, consistent with the extended polyoxyethylene chain length of OP-10 molecules. The improved dispersion state compared to unmodified nanoparticles (Figure S1) confirms the stabilizing effect of surfactant adsorption. Scale bars: (a) 200 nm; (b) 50 nm.

Figure S3. Adsorption Isotherm of OP-10 on SiO_2 Nanoparticle Surfaces

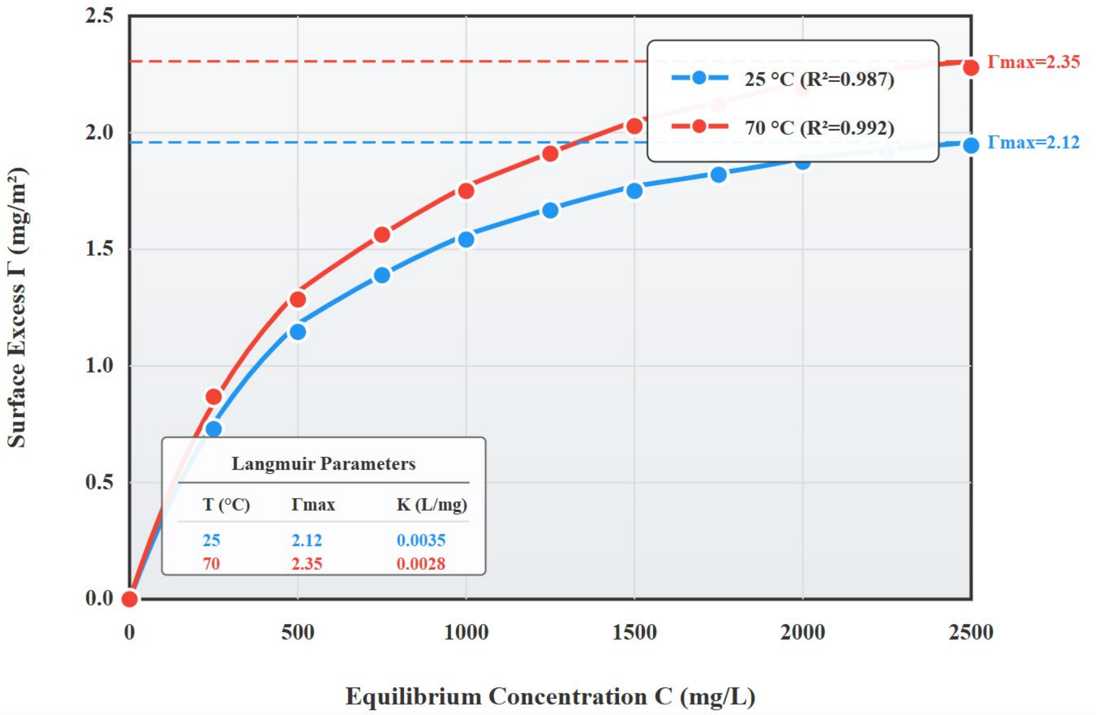

Figure S3. Adsorption isotherms of OP-10 surfactant on SiO_2 nanoparticle surfaces at 25 °C and 70 °C. Experimental data points (symbols) were fitted to the Langmuir isotherm model (solid lines):

$$\Gamma = \frac{\Gamma_{max} \cdot K \cdot C}{1 + K \cdot C}$$

where Γ is the surface excess concentration (mg/m²), Γ_{max} is the maximum adsorption capacity, K is the Langmuir equilibrium constant (L/mg), and C is the equilibrium surfactant concentration (mg/L).

Fitted parameters:

Temperature	Γmax (mg/m²)	K (L/mg)	R^2
25 °C	2.12	0.0035	0.987
70 °C	2.35	0.0028	0.992

The increase in maximum adsorption capacity (Γmax) with temperature indicates enhanced surfactant–nanoparticle interactions at elevated temperatures, attributed to increased molecular mobility and conformational flexibility of polyoxyethylene chains. The relatively high Γmax values suggest effective monolayer surface coverage, which is essential for steric stabilization and hydrogen bonding interactions between OP-10 and SiO_2 surfaces.

Figure S4. Core Sample Photographs

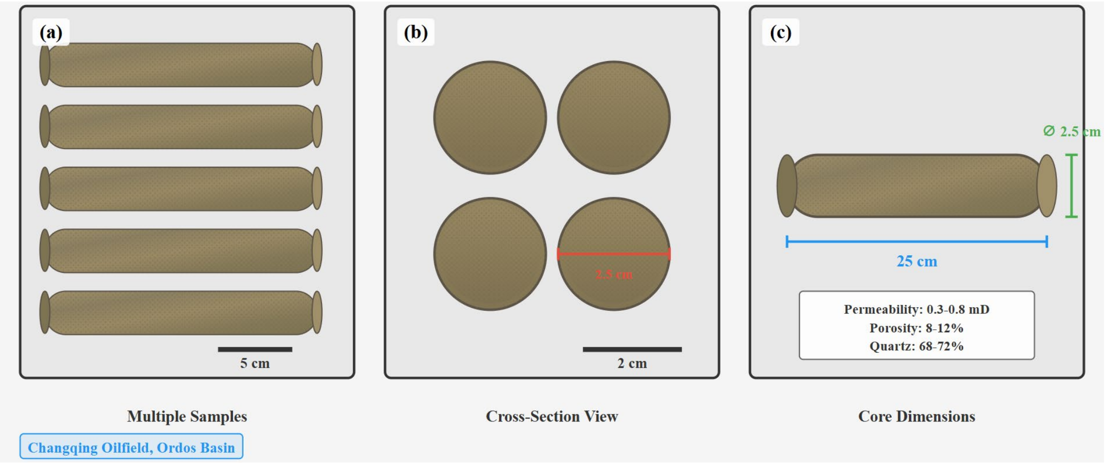

Figure S4. Photographs of natural core samples from the low-permeability sandstone reservoir in Changqing Oilfield used for displacement experiments. (a) Representative core samples after oil washing and drying, showing uniform gray coloration characteristic of tight sandstone lithology; (b) Cross-sectional view demonstrating homogeneous texture without visible fractures, vugs, or macro-heterogeneities; (c) Core samples prepared for displacement experiments with dimensions of 25 cm length × 2.5 cm diameter. The cores exhibit consistent lithological characteristics representative of the Ordos Basin tight sandstone formations, with permeability ranging from 0.3 to 0.8 mD and porosity between 8% and 12%. X-ray diffraction (XRD) analysis confirmed mineralogical composition of quartz (68–72%), feldspar (15–18%), and clay minerals (10–15%, primarily illite and kaolinite).

Experimental Methods for Supplementary Figures

TEM Sample Preparation (Figures S1 and S2)

Nanoparticle suspensions were diluted to 100 mg/L with deionized water. For the composite system, nanoparticles and surfactant were mixed at the 3:2 ratio and stirred for 2 hours before dilution. A drop of each suspension was placed on a carbon-coated copper grid (300 mesh) and

allowed to air dry at room temperature for 24 hours. TEM imaging was performed using a JEOL JEM-2100 transmission electron microscope operated at 200 kV.

Adsorption Isotherm Measurements (Figure S3)

Batch adsorption experiments were conducted by adding 0.5 g of SiO_2 nanoparticles to 50 mL of OP-10 solutions with concentrations ranging from 100 to 3000 mg/L. Suspensions were equilibrated for 24 hours at constant temperature (25 °C or 70 °C) with continuous stirring. After equilibration, samples were centrifuged at 10,000 rpm for 30 minutes, and the supernatant was analyzed for residual surfactant concentration using UV-Vis spectroscopy at 275 nm. The amount of surfactant adsorbed was calculated from the difference between initial and equilibrium concentrations, normalized to the specific surface area of SiO_2 nanoparticles (measured as 180 m^2/g by BET analysis).

Core Sample Documentation (Figure S4)

Core samples were photographed using a Canon EOS 5D Mark IV digital camera under standardized lighting conditions. Samples were cleaned with toluene to remove residual oil, dried at 105 °C for 48 hours, and photographed against a neutral gray background with a calibrated scale bar.
